# Correction: Valenti et al. The miR-205-5p/BRCA1/RAD17 Axis Promotes Genomic Instability in Head and Neck Squamous Cell Carcinomas. *Cancers* 2019, *11*, 1347

**DOI:** 10.3390/cancers17071089

**Published:** 2025-03-25

**Authors:** Fabio Valenti, Andrea Sacconi, Federica Ganci, Giuseppe Grasso, Sabrina Strano, Giovanni Blandino, Silvia Di Agostino

**Affiliations:** 1 Oncogenomic and Epigenetic Unit, Department of Diagnostic Research and Technological Innovation, IRCCS Regina Elena National Cancer Institute, 00144 Rome, Italy; fabio.valenti@ifo.gov.it (F.V.); andrea.sacconi@ifo.gov.it (A.S.); federica.ganci@ifo.gov.it (F.G.); misteri@iol.it (G.G.); 2 Molecular Chemoprevention Group, Department of Diagnostic Research and Technological Innovation, IRCCS Regina Elena National Cancer Institute, 00144 Rome, Italy; sabrina.strano@ifo.gov.it

## Error in Figure 5H

In the original publication [1], there was a mistake in the choice of image for the staining of the RAD17 protein. The image of the RAD17 control sample was mistakenly taken from a slide stained for the BRCA1 expression, as published. We provided the Editorial Office with all the original microscopic acquisitions for each group of staining carried out with BRCA1 and RAD17 antibodies. The corrected Figure 5 appears below. The authors apologize for any inconvenience caused and state that the scientific conclusions are unaffected. This correction was approved by the Academic Editor. The original publication has also been updated.

**Figure 5:**
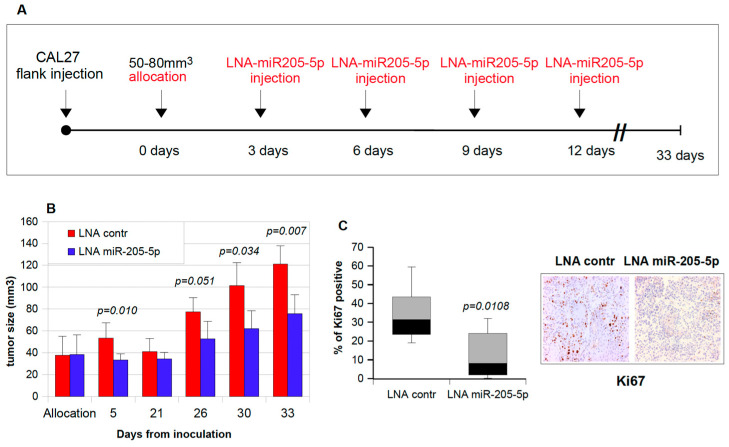

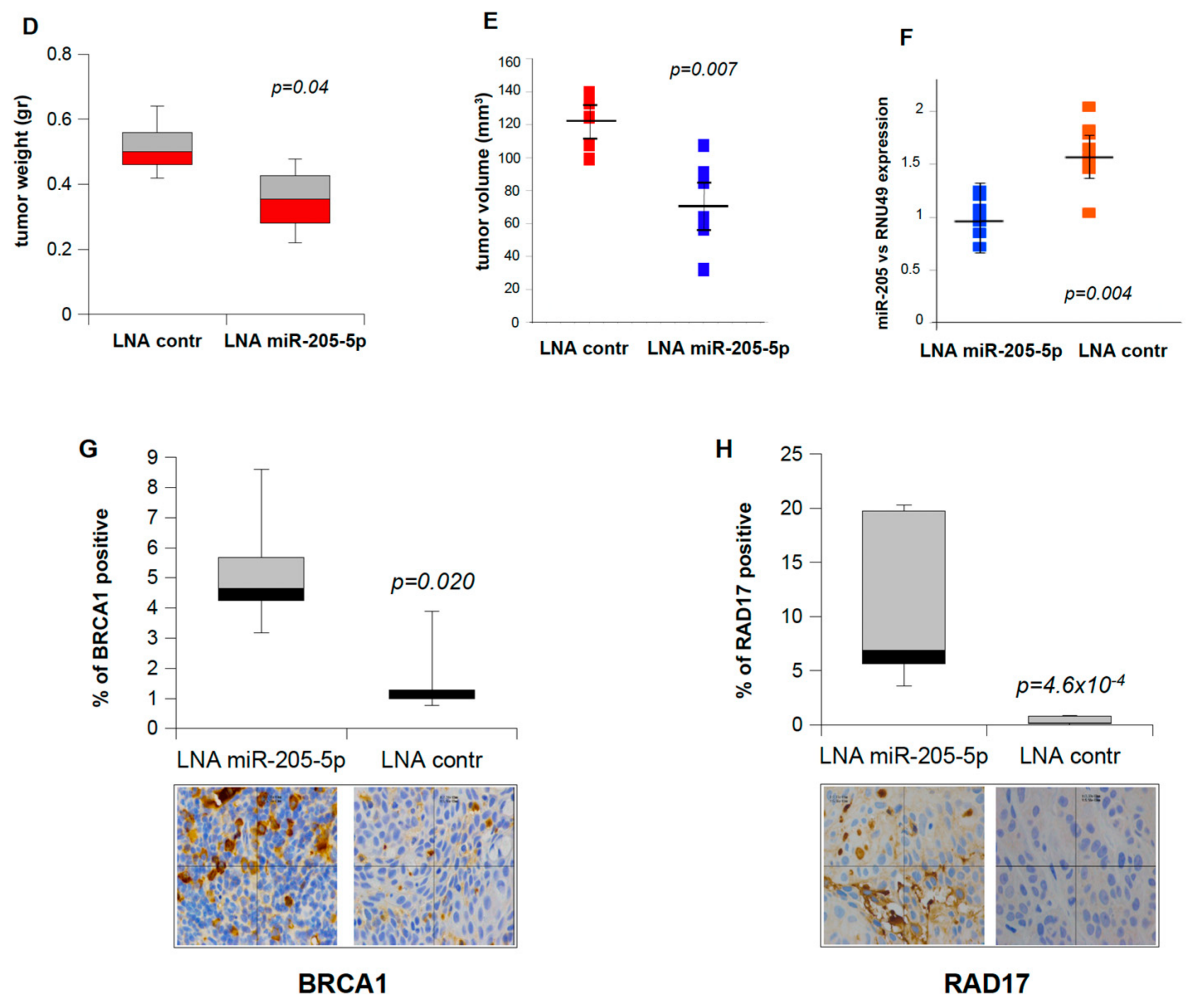
Intratumoral injections of miR-205-5p inhibitor downsize established HNSCC xenografts. (**A**) Schematic overview of in vivo experiments performed on orthotopic HNSCC. The tumor growth was monitored with the aid of a caliper. Mice assigned to the treatment and control groups were treated three times with miR-205-5p inhibitors or corresponding control oligonucleotides every three days; *N* = 6 mice in each group. In each panel, *p*-values were calculated by a two-sample *t*-test. (**B**) A total of 10^6^ CAL27 cells in 30% matrigel were subcutaneously injected into immunodeficient Balb/C mice. On days 3, 6, 9, and 12 following mice allocation, synthetic LNA miR-205-5p inhibitor or control miRNAs conjugated with the Invivofectamine® 3.0 transfection reagent were intratumorally delivered into groups of six animals. Caliper measurements were taken to determine the length and width of each tumor and to calculate total tumor volumes. (**C**) Immunohistochemical analysis of Ki67 protein expression was analyzed in six LNA control- and LNA miR-205-5p-treated mice. Representative images and the relative quantification of ki67 positive cells are shown. (**D**) Tumor weight of the excised tumors measured at the end of the experiment. (**E**) Tumor volumes measured at the end of the experiment. (**F**) Relative miR-205-5p expression in CAL27 tumors. Total RNA was extracted from tumors harvested at the end of the experiment, and RT-qPCR was performed using a specific probe for miR-205-5p (Taq-Man assay). The normalization was carried out using RNU49 throughout the standard curve method. The *p*-values were calculated by a two-sample *t*-test; significant results are marked by a *p*-value <0.05. (**G**,**H**) Immunohistochemistry on tumors treated with LNA control and LNA miR-205-5p. Sections from each mouse were incubated with an anti-BRCA1 antibody (**G**) and anti-RAD17 antibody (**H**). Representative fields are shown.
